# qiRNApredictor: A Novel Computational Program for the Prediction of qiRNAs in *Neurospora crassa*

**DOI:** 10.1371/journal.pone.0159487

**Published:** 2016-07-18

**Authors:** Haiyou Deng, Quan Liu, Wei Cao, Rong Gui, Chengzhang Ma, Ming Yi, Yuangen Yao

**Affiliations:** Department of Physics, College of Science, Huazhong Agricultural University, Wuhan, Hubei, China; CSIR Institute of Genomics and Integrative Biology, INDIA

## Abstract

Recently, a new type of small interfering RNAs (qiRNAs) of typically 20~21 nucleotides was found in *Neurospora crassa* and rice and has been shown to regulate gene silencing in the DNA damage response. Identification of qiRNAs is fundamental for dissecting regulatory functions and molecular mechanisms. In contrast to other expensive and time-consuming experimental methods, the computational prediction of qiRNAs is a conveniently rapid method for gaining valuable information for a subsequent experimental verification. However, no tool existed to date for the prediction of qiRNAs. To this purpose, we developed the novel qiRNA prediction software package qiRNApredictor. This software demonstrates a promising sensitivity of 93.55% and a specificity of 71.61% from the leave-one-out validation. These studies might be beneficial for further experimental investigation. Furthermore, the local package of qiRNApredictor was implemented and made freely available to the academic community at Supplementary material.

## Introduction

Small non-coding RNAs (sRNAs) of 20~30 nucleotides (nt) have gained significant attention in recent years as they are widely involved in various biological processes such as the embryonic, neuronal, muscle, and germline development [1–3]. QDE-2-interacting small RNAs (qiRNAs) of typically 20~21 nt are a new class of sRNAs. qiRNAs are induced by DNA damage [4] and mediate gene silencing in the DNA damage response (DDR) pathway in *Neurospora crassa* by inhibiting protein translation [4]. Although qiRNAs were first discovered in 2009, only little is known about their biogenesis and functionality. It has been demonstrated that the biogenesis of qiRNAs requires DNA-damage-induced aberrant RNAs (aRNAs) as precursors [4]. Moreover, RNA-dependent RNA polymerase QDE-1, the Werner and Bloom RecQ DNA helicase homologue QDE-3, as well as dicers have been previously shown to be involved in the production of qiRNAs [4]. After DNA damage, aRNAs are highly induced and then specifically recognized by RNA-dependent polymerases to produce double-stranded RNAs (dsRNAs). Subsequently, dsRNAs are converted to qiRNAs by dicers [4, 5]. However, the detailed mechanism for the generation of qiRNAs is largely unknown and requires elucidation in further experimental studies.

Previously, novel qiRNAs in *Neurospora crassa* [4] or rice were identified almost exclusively by immunoprecipitation followed by sequencing. However, this conventional approach for the identification of qiRNAs is time-consuming, labor-intensive, and inefficient and even holds the risk of not detecting lowly expressed or issue-specific qiRNAs. Computational methods can overcome these experimental hurdles and extract general information from known qiRNAs to predict novel qiRNAs for further experimental manipulation [6]. However, as far as we know, no qiRNA prediction tool has been implemented so far. Therefore, the development of efficient computational approaches for qiRNA prediction is urgently needed and intriguing.

Common sequence or structure conservation-based approaches in microRNAs (miRNAs) prediction cannot be directly adopted for qiRNA prediction as no evidence has been reported for a high evolutionary conservation of the sequence and structure of qiRNAs across species. Unlike Piwi-interacting RNAs (piRNAs), the density of plot of qiRNAs does not show a striking clustering characteristic [4]. Thus, no clustering characteristic can be used for qiRNA prediction. However, it has been demonstrated that qiRNAs exhibit strong position-specific preferences for uracil (U) at the first nucleotide of the 5’ end and for adenine (A) at the first nucleotide of the 3’ end [4]. In bioinformatics, position-specific nucleotide preferences are usually employed for sRNAs prediction. For example, ping-pong-dependent piRNAs show some position-specific nucleotide preferences, such as for thymine (T) at the first position (1T) and for A at the 10th position (10A) [6]. To capture and leverage these sequence features for piRNA prediction, Betel *et al*. constructed a 21 × 4 feature vector based on a 21-base window around the 5’ end (plus 10 nt upstream and 10 nt downstream) and trained a support vector machine (SVM) model for piRNA prediction [7]. In 2011, Zhang *et al*. used a simple *k*-mer scheme to construct 1364 dimension feature vectors for describing the candidate piRNA sequences and then constructed the software piRNApredictor for piRNA prediction based on an improved Fisher linear algorithm [6]. Moreover, position-specific scoring matrix (PSSM) is a commonly used representation of sequence motifs, which has been widely applied to the prediction of significant biological signals such as DNA binding sites [8], RNA binding sites [9], promoters [10], protein secondary structure prediction [11], etc.

In this work, we developed an ingenious method for the extraction of features based on position probability matrices (PPM) of biosequences and then constructed a novel qiRNApredictor (qiRNA predictor) software package with 80 features. Firstly, the experimentally verified qiRNAs were collected manually from the scientific literatures and then used for training the Random forest (RF) model using the randomForest R package. Subsequently, the performance and robustness of qiRNApredictor were extensively evaluated by *n*-fold cross-validations (CV) as well as the leave-one-out cross-validation (LOO-CV). Upon LOO-CV, qiRNApredictor exhibits a promising sensitivity of 93.55% and a specificity of 71.61%, which can be used for the prediction of qiRNAs.

## Materials and Methods

### Data preparation

Herein, only qiRNA sequences in *Neurospora crassa* were considered (no qiRNA in rice was sequenced in the work by Chen *et al*. [5]). Lee *et al*. identified 184 individual qiRNA sequences in *Neurospora crassa* [4]. However, 23 qiRNA sequences were directly discarded as they included undefined bases. We further eliminated six individual qiRNA sequences as they were identical to other qiRNA sequences. Finally, 155 experimentally verified qiRNAs were collected as positive samples to train a RF model. Three negative datasets were built. For the convenience of discussion, we called them the “Random”, the “sRNA-segment”, and “milRNA” negative datasets, according to the ways we collected them or the data origin. The “Random” negative dataset was randomly extracted from NONCODE database. As previously described [6], non-coding RNA sequences obtained from the NONCODE database (version 3.0) [12] were fragmented into non-overlapping segments. For each of these non-qiRNA segments, we shuffled it 10000 times to destroy any potentially functional structures [6]. Then, the same amount of negative samples were randomly selected from the segments under the constraint condition that the length distribution of the selected segments was identical with that of positive qiRNAs. The “sRNA-segement” negative dataset was collected from Rfam database. The sequences of sRNAs of *Neurospora crassa* were retrieved from Rfam database (release 12.0) [13]. Because the lengths of sRNAs are much longer than that of qiRNAs, we fragmented the sequences of sRNAs into non-overlapping segments under the constraint that the length distribution of sRNA segments was similar to that of positive qiRNAs. Then, the same amount of sRNA segments were randomly selected and regarded as the analogue of the degraded fragments of sRNAs. miRNA-like small RNAs (milRNA) are about 19~25 nt and have a strong preference (51.08%) for U at the 5’ end. They were firstly discovered in *Neurospora crassa* and called milRNAs due to their similarities with miRNAs [14]. Furthermore, 325 milRNAs were manually collected from the scientific literatures to construct the “milRNA” negative dataset. Since only 25 milRNAs were obtained from *Neurospora crassa*, we also collected the putative milRNAs from other fungi species, including *Trichoderma reesei* [15], *Sclerotinia sclerotiorum* [16], *Metarhizium anisopliae* [17], *Zymoseptoria tritici* [18], *Fusarium oxysporum* [19], *Fusarium graminearum* [20], *Antrodia cinnamomea* [21], *Aspergillus flavus* [22], *Penicillium chrysogenum* [23], and *Penicillium marneffei* [24]. Finally, these three negative datasets were incorporated with the positive dataset respectively to construct the “Random”, “sRNA-segment” and “milRNA” training datasets.

### Feature extraction

**Fig 1:**
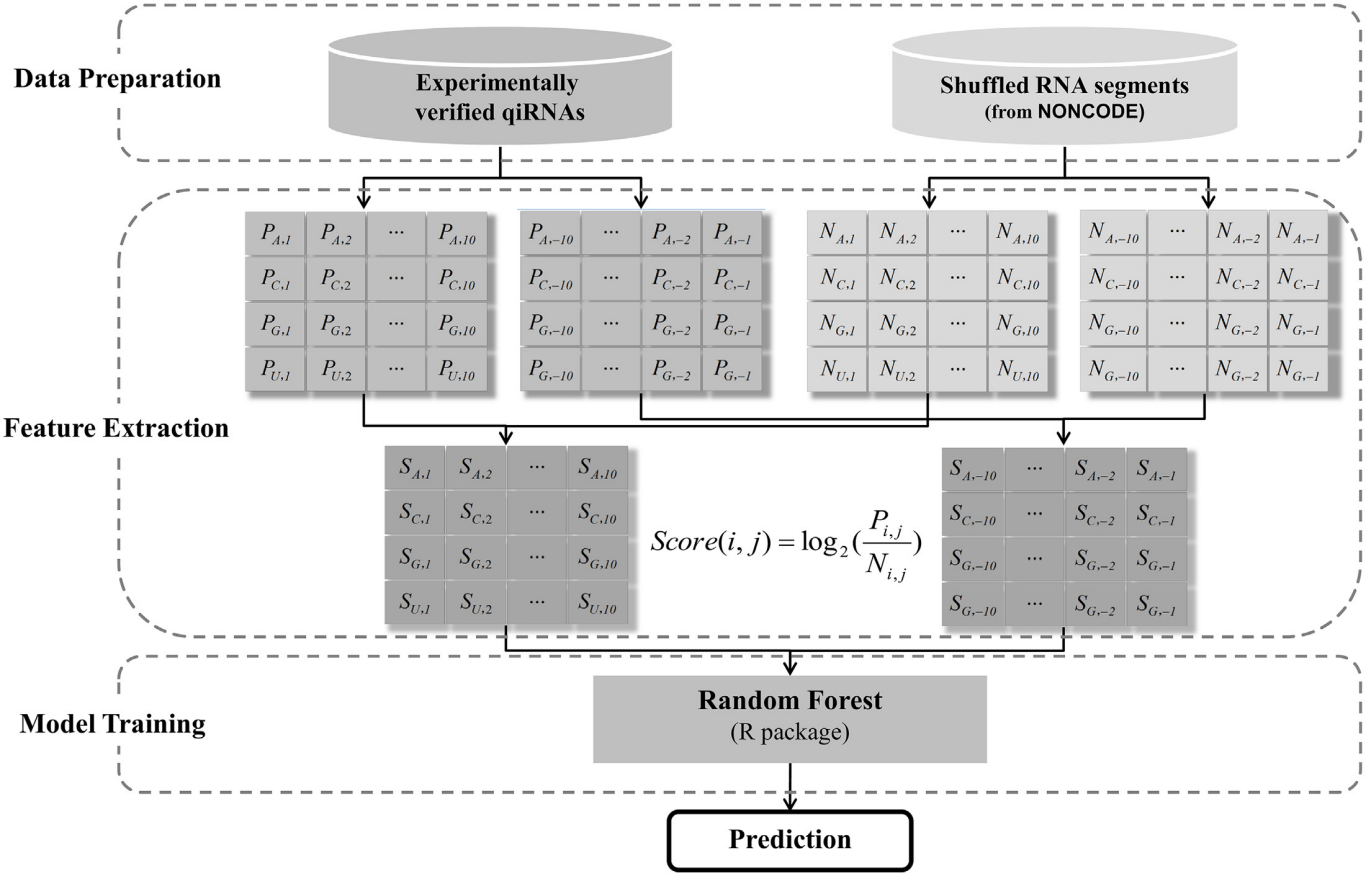
The flowchart of qiRNApredictor.

The extraction of an appropriate set of features for training a prediction model is one of the vital, yet most challenging issues in machine learning-based prediction approaches. To capture the position-specific preference of nucleotides, the occurrence probabilities of each nucleotide at the first ten positions (1 through 10) and the last ten positions (-1 to -10) of both positive and negative samples were calculated to create four PPMs (Fig 1). It should be noted that qiRNA sequences of less than 20 nt exhibit some overlaps of the first and last ten positions. The score of nucleotide *i* at position *j* can be calculated according to the formula

$$\text{S}\left(i,j\right)={\text{log}}_{2}\left(\frac{P_{ij}}{N_{ij}}\right),\;i\in \left[\text{A},\text{C},\text{G},\text{U}\right],\;j\in \left[-10,\cdots ,-1;\;1,\cdots ,10\right]$$

where ***P***_*ij*_ and ***N***_*ij*_ are the probability of nucleotide *i* at position *j* in PPMs produced by positive and negative samples, respectively (Fig 1). The score gives an indication how much the position-specific preference of positive samples differs from that of negative samples. For the cases with ***P***_*ij*_ = ***N***_*ij*_, the score equals zero, which means this feature cannot offer any information for the prediction. However, when ***P***_*ij*_ or ***N***_*ij*_ is zero, it doesn’t mean this feature has great significance. Instead, it may be simply because of the limited sample size. To circumvent the infinite value caused by inadequate sampling, we assigned a zero score for the cases that ***P***_*ij*_ or ***N***_*ij*_ is zero. In total, 80 scoring values were directly regarded as features to train a RF model. Then *F*-score was used to measure the discriminatory power of each feature above [25]. The *F*-score of the *i*th feature is defined as:

$$F\left(i\right)\equiv \frac{{\left({\bar{x}}_{i}^{\left(+\right)}-{\bar{x}}_{i}\right)}^{2}+{\left({\bar{x}}_{i}^{\left(-\right)}-{\bar{x}}_{i}\right)}^{2}}{\frac{1}{n_{+}-1}\sum_{k=1}^{n_{+}}{\left(x_{k,i}^{\left(+\right)}-{\bar{x}}_{i}^{\left(+\right)}\right)}^{2}+\frac{1}{n_{-}-1}\sum_{k=1}^{n_{-}}{\left(x_{k,i}^{\left(-\right)}-{\bar{x}}_{i}^{\left(-\right)}\right)}^{2}}$$

where *n*_+_ and *n*_−_ are the numbers of positive and negative samples, respectively; ${\bar{x}}_{i}$, ${\bar{x}}_{i}^{\left(+\right)}$, and ${\bar{x}}_{i}^{\left(-\right)}$ are the average of the *i*th feature of total, positive, and negative samples, respectively. $x_{k,i}^{\left(+\right)}$ and $x_{k,i}^{\left(-\right)}$ are the *i*th feature of the *k*th positive and negative sample, respectively. Larger *F*-scores indicate better discrimination [25].

### Random forest

RF is an ensemble learning classifier consisting of a multitude of tree-structured classifiers [26]. RF makes use of two powerful machine-learning methods—the bagging and random feature selection in the tree induction [27]. In the bagging algorithm, each tree is trained by using a bootstrap sample of the training data, and the prediction results of the ensemble are aggregated by majority vote or averaging rule to give the final prediction results [27]. Instead of using all features, only a random subset of features was used here to split at each node when growing a single tree. A type of CV together with the training step using out-of bag (OOB) samples was used to assess the prediction performance of RF. More specifically, a particular bootstrap sample was adopted to grow each tree during the process of training. Since bootstrapping sampling is sampling with replacement, some of the samples were ignored, while others were reused. The ignored samples constitute the OOB sample. On average, 1−*e*^−1^ ≅ 2/3 of the training samples was used for growing the tree leaving *e*^−1^ ≅ 1/3 as OOB, which have not been used for tree construction. Therefore, these samples could be used to evaluate the prediction performance [26, 27]. The RF algorithm was implemented by the randomForest R package.

### Performance evaluation

As previously described [28], among the predicted positive results obtained by qiRNApredictor, the real positives are called true positives (*TP*), while the other positive results are called false positives (*FP*). Among the predicted negative results obtained by qiRNApredictor, real negatives are called true negatives (*TN*), while the other negative results are called false negatives (*FN*). The performance is evaluated based on four measurements of specificity (*Sp*), sensitivity (*Sn*), accuracy (*Ac*), and Matthew’s correlation coefficient (*MCC*). These indexes are defined as

$$Sn=\frac{TP}{TP+FN},\;Sp=\frac{TN}{TN+FP},\;Ac=\frac{TP+TN}{TP+FP+TN+FN}$$

and

$$MCC=\frac{\left(TP\times TN\right)-\left(FN\times FP\right)}{\sqrt{\left(TP+FN\right)\times \left(TN+FP\right)\times \left(TP+FP\right)\times \left(TN+FN\right)}}$$

In this study, we performed 4-, 6-, 8-, and 10-fold CVs as well as the LOO-CV. Receiver Operating Characteristic (ROC) curves were plotted for performance visualization.

## Results

### A novel algorithm for qiRNA prediction

In this work, we collected experimentally identified qiRNAs from the scientific literatures. After eliminating redundancy in sequences and directly removing sequences that include undefined bases, a dataset of 155 experimentally verified qiRNA sequences was obtained (S1 Table). As previously described, a negative dataset containing 155 samples was constructed. Finally, a balanced database containing 155 positive and negative samples was used for model construction.

**Fig 2:**
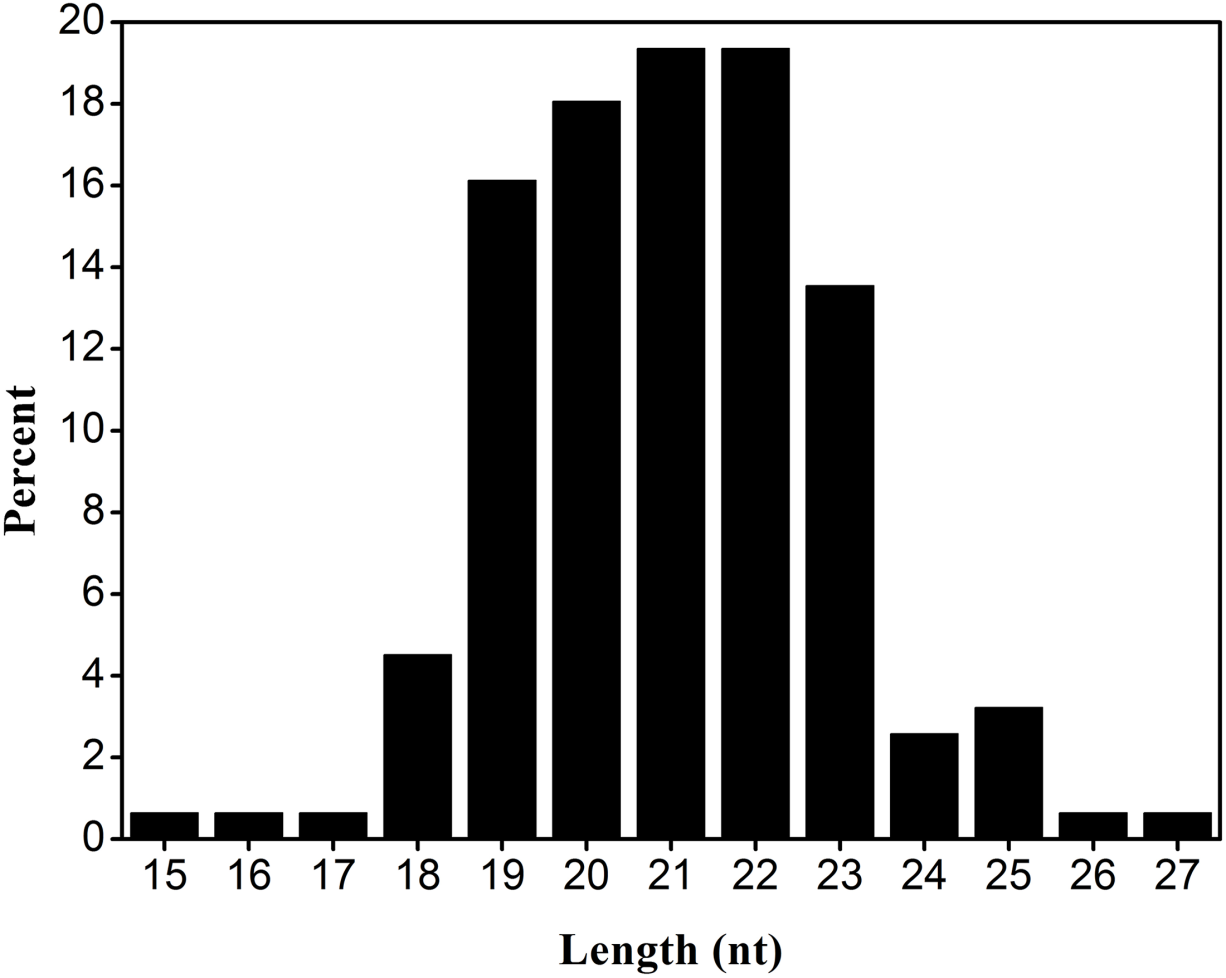
The length distribution of qiRNAs in *Neurospora crassa*.

**Fig 3:**
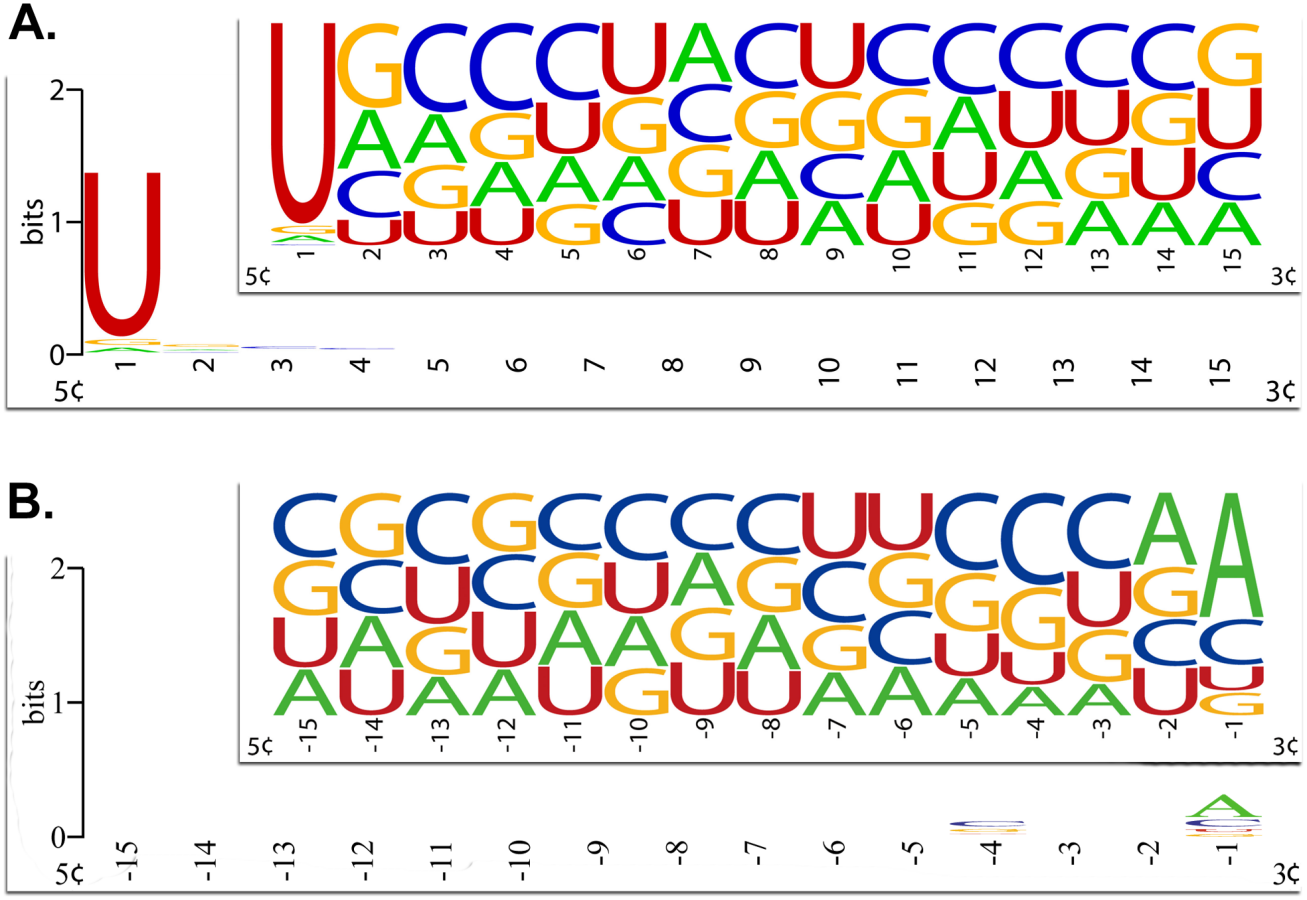
Position-specific nucleotide preferences of qiRNAs in *Neurospora crassa* for (A) the first 15 nt substring from 5’ end to 3’ end and (B) the last 15 nt substring from 5’ end to 3’ end. The insets are Frequency Plot of qiRNA sequences. The sequence logo analysis was implemented by WebLogo [29].

**Fig 4:**
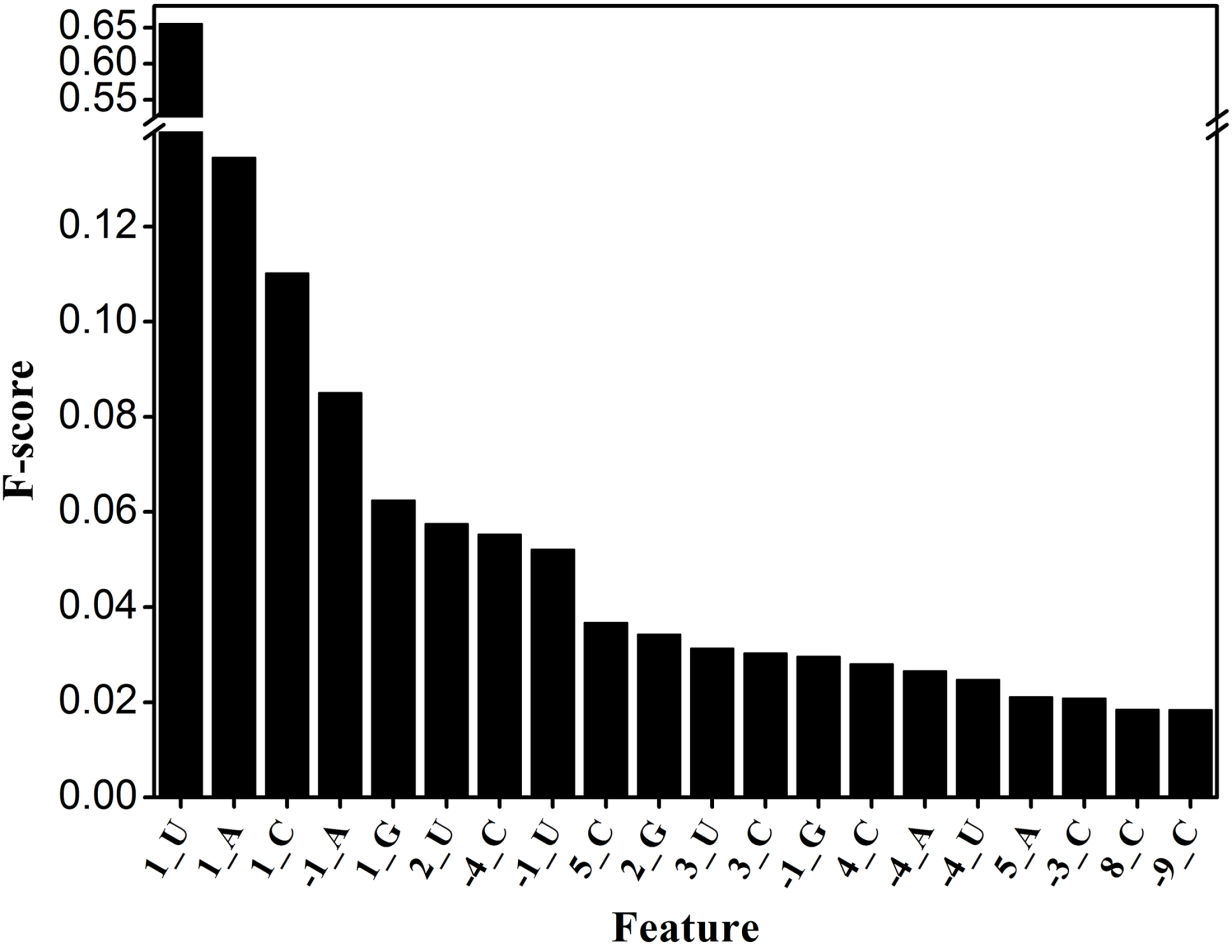
The *F*-scores of PPM features. Larger *F*-scores indicate better discrimination.

qiRNAs are very short of approximately 20~21 nt in length (Fig 2), which renders it very difficult to predict qiRNAs with high accuracy. However, the first and last nucleotide of qiRNAs have a high preference for U and A, respectively (Fig 3). Therefore, we attempted to predict qiRNAs with the position-specific preferences of nucleotides in qiRNA sequences. To this goal, PPMs were firstly constructed for characterizing position-specific properties of qiRNAs. Based on the PPMs, 80 log-likelihood scores were calculated as features for training prediction model (see details in Materials and Methods). Based on the training datasets, we used the *F*-score [25] to rank 80 features. As expected, nucleotides at the first and last position, such as 1U, 1A, 1C, or -1A, exhibit high *F*-scores (Fig 4). This is consistent with the position-specific preferences of nucleotides in qiRNA sequences, which demonstrates that we have succeeded in capturing these characteristics in qiRNA sequences.

**Fig 5:**
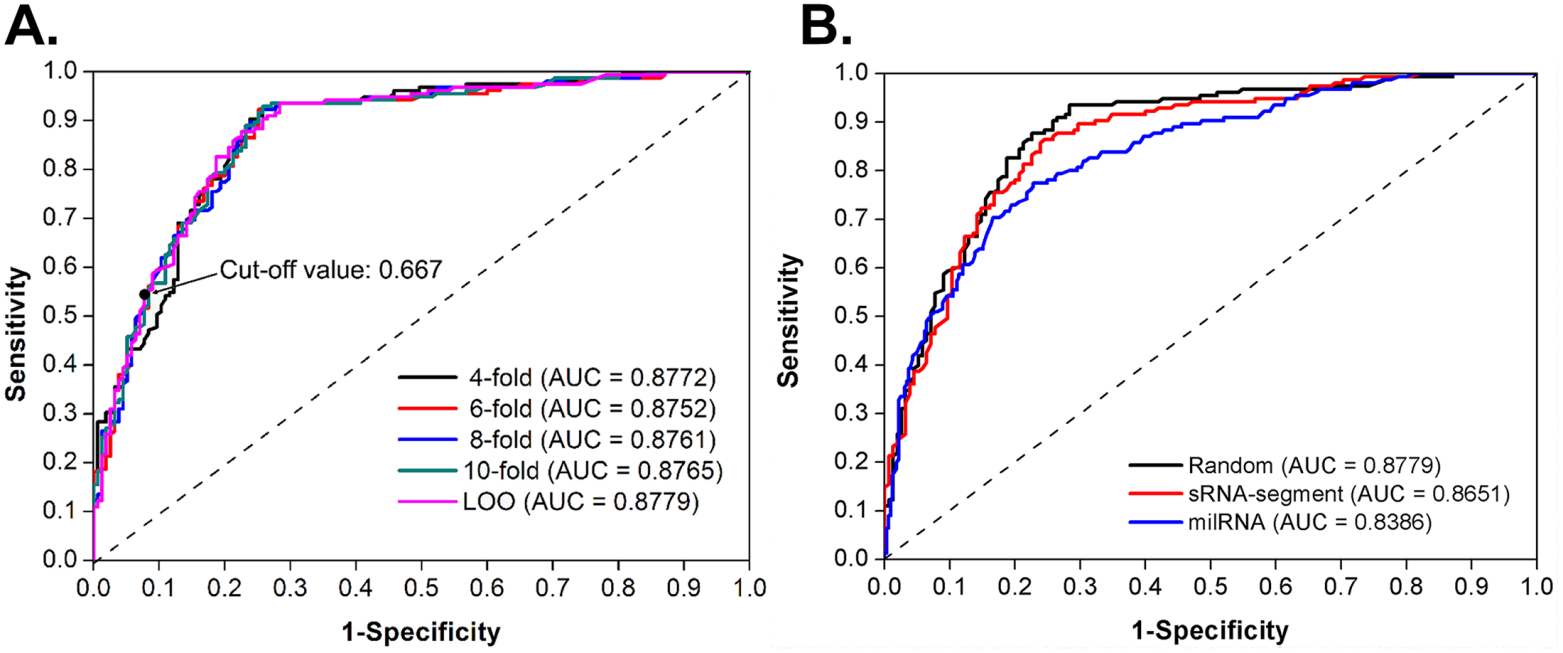
The prediction performance of qiRNApredictor. (A) The LOO-CV as well as 4-, 6-, 8-, and 10-fold CVs based on “Random” training dataset were calculated. The ROC curves and AUCs were also drawn and analyzed. To increase the specificity, we recommended a stringent cut-off value 0.667 for experimental investigation. The specificity of qiRNApredictor with the cut-off value 0.667 is 92.26%, while the sensitivity is 54.84%. (B) The ROC curves and AUCs were drawn and analyzed for the “Random”, “sRNA-segment”, and “milRNA” training datasets, respectively, by LOO-CV.

To evaluate the performance and robustness of the qiRNApredictor, LOO-CV and 4-, 6-, 8-, and 10-fold CVs were performed. The LOO-CV results based on “Random” training dataset show that our method predicts at 93.55% sensitivity, 71.61% specificity, 82.58% accuracy and 0.6679 *MCC* value (Table 1). The results of the 4-, 6-, 8-, and 10-fold CVs are also close to those of the LOO-CV. From the ROC curves (Fig 5A), AUC (area under ROC curves) values were calculated as 0.8779 (LOO-CV), 0.8772 (4-fold CV), 0.8752 (6-fold CV), 0.8761 (8-fold CV), and 0.8765 (10-fold CV), respectively (Fig 5A). As no qiRNA prediction tool existed to date, the performance of our qiRNA prediction tool could not be compared to any existing tool. To further test the ability of qiRNApredictor in distinguishing qiRNAs among other sRNAs, we also run it through “sRNA-segement” and “milRNA” datasets. milRNA is 19~25 nt in length with a strong preference (51.08%) for U at the 5’ end, which is somewhat similar to qiRNA. As shown in Fig 5B & Table 1, the results demonstrate that the qiRNApredictor is able to identify qiRNAs among other sRNAs with quite significant AUC, *MCC*, sensitivity and specificity. Taken together, qiRNApredictor is a promising tool for the prediction of candidate qiRNAs.

**Table 1:** The performances of qiRNApredictor on each training dataset by the LOO CV.

Dataset	*Ac* (%)	*Sn* (%)	*Sp* (%)	*MCC*
**“Random” training dataset**	82.58	93.55	71.61	0.6679
**“sRNA-segement” training dataset**	80.97	86.45	75.48	0.6231
**“milRNA” training dataset**	79.17	70.32	83.38	0.5303

### Comparison to k-mer feature classes

**Fig 6:**
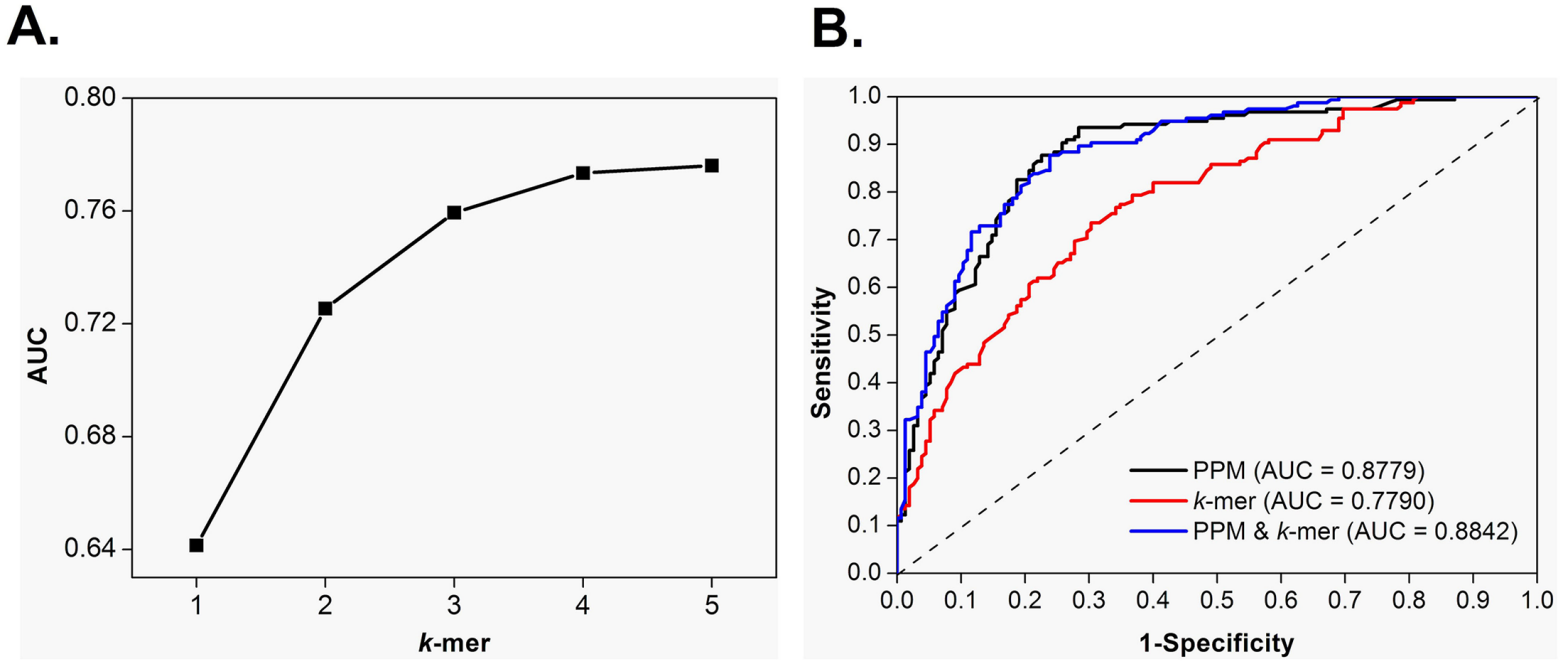
Comparison to *k*-mer feature classes. (A) Effect of the *k* parameter in *k*-mer feature classes on the prediction performance by 5-fold CV; (B) The ROC curves for RFs trained with different feature classes.

In bioinformatics, *k*-mers usually refer to *k*-gram or *k*-tuples of DNA or protein sequences and can be used to find certain regions within biosequences or be employed as *k*-mer statistics for giving discrete probability distributions of possible *k*-mer combinations [6]. *K*-mers can be used to distinguish qiRNA from non-qiRNA based on differences of string usages between the different sequence classes. Here, 1–5 nt strings were used to characterize positive or negative sequences by a vector consisting of the frequencies of *k*-mer strings. We first adjusted the *k* parameter in *k*-mer feature classes to obtain a better performance based on the same training dataset. With increasing *k* values, the AUC values exhibit first a rapid increase, which slows down in the following (Fig 6A). Although the increase of features may result in over-fitting, the best performance (AUC = 0.7790) of *k*-mer feature classes is lower than that of PPM features classes (AUC = 0.8779) (Fig 6B). This demonstrates that PPM is more suitable for qiRNA prediction. Moreover, we combined PPM and *k*-mer feature classes for the prediction of qiRNAs. However, the prediction performance of the combined feature classes exhibits to be very close to that of single PPM feature classes (Fig 6B).

## Discussion

As a new regulatory factor for mediating gene silencing in the DNA damage response, qiRNAs have increasingly attracted considerable interest and investigative efforts. The identification of qiRNAs is fundamental for dissecting regulatory functions and molecular mechanisms. Compared to other expensive and time-consuming experimental methods, the computational prediction of qiRNAs is a conveniently rapid method of getting useful information for subsequent experimental verification. However, no qiRNA prediction tool existed to date. Therefore, developing a novel approach for qiRNA prediction is required and very intriguing. In this work, we designed a novel software named qiRNApredictor based on PPM features and RF algorithm. The performance and robustness of qiRNApredictor were extensively evaluated by n-fold CVs as well as LOO-CV, which gave very promising results.

**Fig 7:**
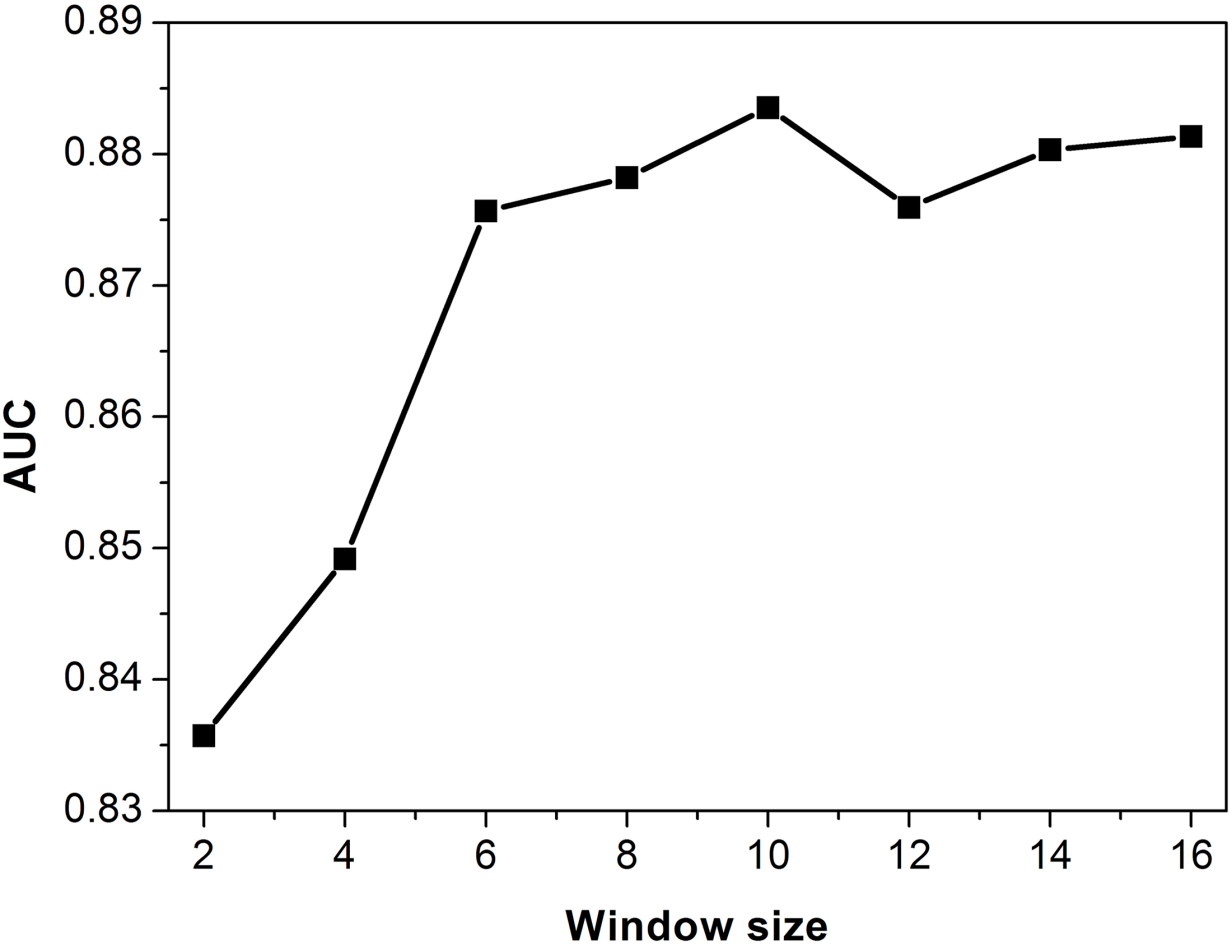
Effect of the window size in PPM feature classes on the prediction performance by 5-fold CV.

Here, the window size refers to the length of the region of interest at both ends of the sequences. To evaluate the effect of window size on the prediction performance, the window size was adjusted, and the AUC value was calculated based on the results of 5-fold CV. The AUC values first rapidly increase and then slow down with an increasing window size (Fig 7). There is a qiRNA sequence of only 15 nt, which was simply ignored when testing the window of 16 nt. Interestingly, the combination of two adjacent bases at both ends of sequences obtained a high AUC value of 0.8357. This either demonstrates that the characteristic signal at both ends of the qiRNA sequences is very significant, or it suggests that non-qiRNAs are not very similar to genuine qiRNAs. However, the approach of non-qiRNAs construction is standard according to previous studies [6].

The performance of a classifier system would be improved by selecting the most discriminative set of features due to reducing the complexity of the classifier system. In contrast, the simple combination of PPM and *k*-mer feature classes does not improve the performance of the classifier system. This suggests that the feature set exhibits redundancies, which leaves room for optimization of the feature set used in qiRNApredictor in the subsequent work. Furthermore, the prediction performance of qiRNApredictor would be improved by finding the secondary structure features of qiRNA precursors. Taken together, our studies provide a novel and promising approach for qiRNA prediction and will facilitate further functional studies of qiRNAs.

## Supporting Information

S1 File The compressed files in ZIP format of the local package of qiRNApredictor. (ZIP)

S1 Table The dataset of 155 experimentally verified qiRNAs in *Neurospora crassa* obtained from the work by Lee *at al* [4]. (XLS)
