# Supplementary material for: qiRNApredictor: A Novel Computational Program for the Prediction of qiRNAs in Neurospora crassa
Source: PLoS One. 2016 Jul 18;11(7):e0159487. doi: 10.1371/journal.pone.0159487 (PMC4948831; doi:10.1371/journal.pone.0159487)
Supplement: S1 File — (ZIP) [file pone.0159487.s001.zip › qiRNApredictor_Linux/README.htm]

qiRNApredictor User Manual


## qiRNApredictor User Manual

Apr 7 2015

---

### About This Program

Recently, a new type of small interfering RNAs (qiRNAs) of typically 20~21 nucleotides were found in Neurospora crassa and rice,
and have been shown to regulate gene silencing in the DNA damage response. Identiﬁcation of qiRNAs is fundamental for dissecting
the regulatory functions and molecular mechanisms. In contrast with expensive and time-consuming experimental methods,
the computational prediction of qiRNAs is a conveniently rapid method of getting useful information for subsequent experimental veriﬁcation.
However, no available tool was made for qiRNA prediction. Here we developed a novel software package qiRNApredictor to predict qiRNAs.
The software demonstrated a promising sensitivity of 93.55% and a specificity of 71.61% from the leave-one-out validation.
These studies might be helpful for further experimental investigation.

---

#### Usage:

qiRNApredictor [options]

#### options:

|  |  |
| --- | --- |
| -D | The result directory |
| -P | fasta file containing positive sequences |
| -N | fasta file containing negative sequences |
| -T | fasta file containing test sequences |
| -h | print help information |

#### example:

qiRNApredictor -D result -P positive.fa -N negative.fa -T test.fa

---

### System Requirements

Windows versions:  
Any 32 bit Windows system. E.g. Windows 95/98/NT/2000/ME/XP/2003, etc

Unix versions:  
Solaris, Linux, etc, or any 32 bit Windows system. E.g. Windows
95/98/NT/2000/ME/XP/2003 , etc

---

### Prerequisites

Please ensure R software and R package of "randomForest" are installed before proceeding.

You can download the installation file of R software from http://www.cran.r-project.org/.

Then follow the installation prompts of R software to finish installation.

---

### Installation - Win32 versions

Unzip the installation file and add the directory to your path.

---

### Availability

Our softwares are freely available for academic researches.
For non-profit users, you can copy, distribute and use the softwares for your scientific studies.
Our softwares are not free for commercial usage.

For ordering information please please contact us.

---

### Citation

If the software has been helpful for your work, we wish you could cite the article.

---

### Contacting the Author

We'd like to hear your questions and comments!

If you are having trouble getting your executable to run, please contact Dr. Yuangen Yao by E-mail: yyg@mail.hzau.edu.cn

Department of Physics, College of Science, Huazhong Agricultural University, Wuhan, Hubei 430070, China

---
